# Measuring Pre- and Post-Copulatory Sexual Selection and Their Interaction in Socially Monogamous Species with Extra-Pair Paternity

**DOI:** 10.3390/cells10030620

**Published:** 2021-03-11

**Authors:** Emily Rebecca Alison Cramer

**Affiliations:** Sex and Evolution Research Group, Natural History Museum, University of Oslo, 0318 Oslo, Norway; becky.cramer@nhm.uio.no

**Keywords:** pre-copulatory sexual selection, post-copulatory sexual selection, mating systems, passerines, extra-pair paternity, sperm competition, cryptic female choice, evolution, fertilization, reproduction, gametes

## Abstract

When females copulate with multiple males, pre- and post-copulatory sexual selection may interact synergistically or in opposition. Studying this interaction in wild populations is complex and potentially biased, because copulation and fertilization success are often inferred from offspring parentage rather than being directly measured. Here, I simulated 15 species of socially monogamous birds with varying levels of extra-pair paternity, where I could independently cause a male secondary sexual trait to improve copulation success, and a sperm trait to improve fertilization success. By varying the degree of correlation between the male and sperm traits, I show that several common statistical approaches, including univariate selection gradients and paired t-tests comparing extra-pair males to the within-pair males they cuckolded, can give highly biased results for sperm traits. These tests should therefore be avoided for sperm traits in socially monogamous species with extra-pair paternity, unless the sperm trait is known to be uncorrelated with male trait(s) impacting copulation success. In contrast, multivariate selection analysis and a regression of the proportion of extra-pair brood(s) sired on the sperm trait of the extra-pair male (including only broods where the male sired ≥1 extra-pair offspring) were unbiased, and appear likely to be unbiased under a broad range of conditions for this mating system. In addition, I investigated whether the occurrence of pre-copulatory selection impacted the strength of post-copulatory selection, and vice versa. I found no evidence of an interaction under the conditions simulated, where the male trait impacted only copulation success and the sperm trait impacted only fertilization success. Instead, direct selection on each trait was independent of whether the other trait was under selection. Although pre- and post-copulatory selection strength was independent, selection on the two traits was positively correlated across species because selection on both traits increased with the frequency of extra-pair copulations in these socially monogamous species.

## 1. Introduction

The potential for sexual selection to occur in the events leading up to copulation has long been recognized [1,2]. Since Parker’s seminal paper 50 years ago [3], the existence of post-copulatory sexual selection has also been acknowledged, with mechanisms ranging from altering the duration of copulation, to altering the amount of parental care provided, depending on characteristics of the copulation partner [4,5]. More recently still, research has begun to examine the interaction between the pre-copulatory episode of sexual selection that culminates in copulation and the post-copulatory episode that culminates in fertilizing the egg [6,7,8,9,10,11]. If the two episodes work synergistically, they can strengthen overall selection; or if they work in opposing directions, the action of one episode may diminish the strength of selection in the other episode [9,12,13]. Moreover, it has been suggested that the post-copulatory episode will be generally weaker, and constrained by, the pre-copulatory episode, since only males that succeed in acquiring copulations can be exposed to post-copulatory sexual selection [14,15,16]. Corroborating this hypothesis, males invest less in post-copulatory traits in species where a male can prevent other males from achieving copulations, and thus prevent their sperm from facing competition [9,17,18]. However, there is substantial variation in whether traits involved in pre- and post-copulatory selection correlate positively or negatively across individuals [13,19] and across species [20,21]; reviewed in [9]. Thus, additional work is needed to explore the interaction between pre-copulatory and post-copulatory sexual selection, across a range of mating systems [9]. 

An important step towards understanding the interaction between selective episodes is to determine which traits are under selection in each episode. For wild populations and internally fertilizing species, directly observing copulations and fertilizations is often not feasible, so it is common to instead infer copulation and fertilization patterns from genetic parentage data. This practice can introduce substantial bias for metrics of pre-copulatory sexual selection, such as the Bateman gradient and opportunity for sexual selection [22]. Even in lab studies where copulations are controlled, early embryo mortality can cause biased estimates of fertilization success from parentage data [23]. Given the great diversity of post-copulatory processes that can impact fertilization success and offspring production following copulations [5], using parentage data to infer selection on sperm traits likely results in bias. However, this question has not been rigorously examined. 

One group of animals where methodological restrictions may have a substantial impact is socially monogamous species with extra-pair paternity. While this mating system occurs in a range of animals [24,25,26,27], it is best studied in passerine birds [28]. Comparative studies have found strong evidence of post-copulatory selection on sperm due to extra-pair paternity. For example, in species with higher levels of extra-pair paternity, sperm morphology is less variable among and within males [29,30,31,32,33], sperm cells are longer and length evolves more quickly [34,35,36], and sperm cells swim more quickly [37]; but see [36]. Laboratory studies where copulations are experimentally controlled similarly show that longer and faster-swimming sperm have higher fertilization success [38,39]. In contrast, field studies on wild populations often find no evidence of selection on sperm traits [40,41,42,43,44]. Potentially, the lack of patterns in these field studies is because post-copulatory sexual selection is not sufficiently strong in the species studied to result in statistical power, and indeed, in some species with high levels of extra-pair paternity, selection on sperm has been detected [45,46]. Alternatively, inferring copulation and fertilization success from genetic parentage may introduce statistical biases that obscure underlying patterns of selection on sperm [47]. Such bias appears particularly likely if male traits involved in copulation success correlate with sperm traits involved in fertilization success (e.g., [44]). 

The first aim of this study was therefore to evaluate the statistical robustness of different analytical approaches for studying selection on male and sperm traits in field studies of socially monogamous animals with extra-pair paternity. The second aim was to explore whether the two episodes of selection interacted synergistically. In simulated populations, I independently varied whether the pre-copulatory and post-copulatory episodes of selection were active, i.e., whether a male trait affected copulation success and whether a sperm trait affected fertilization success. I created populations where only one, or both, episodes of selection were active and with varying correlation between the male and sperm traits. I then used a range of typical analytical approaches to test for selection on each trait (Aim I). Most analytical approaches showed substantial bias for detecting selection on sperm traits. Using an unbiased analytical approach, I tested whether selection on each trait differed depending on whether selection was also active on the other trait, and found no evidence for such an effect (Aim II). However, across species, the strength of selection on male and sperm traits were positively associated. 

## 2. Materials and Methods

### 2.1. Overview

Simulations were based on 15 species of passerine birds, which include much of the range of extra-pair paternity rates observed in the group (Table 1, range in socially monogamous passerines 0–68.5%, [28]; raw data available at [48]). The simulated populations are intended to represent a range of “typical” behaviors for passerines, rather than precise conditions for individual species. Therefore, all simulated populations performed extra-pair copulations only with first- and second-degree neighbors, as is common in many species [49] and references therein). Results for simulation conditions without this spatial constraint are presented in the supplement.

**Table 1:** Empirical values for extra-pair offspring (EPO) and broods containing at least one EPO (EPB) in the 15 songbird species simulated. Estimated values of parameters used in the simulation represent the average number of extra-pair copulations per female (*m*) and the relative number of extra-pair sperm per copulation (*s*).

Species, Citation for Empirical Data	% EPO	% EPB	*m*	*s*	N Broods
Banded wren (*Thryophilus pleurostictus*) [50] and unpublished (ERAC, Michelle Hall, Sandra Vehrencamp)	4	10	0.36	0.27	50
Pied flycatcher (*Ficedula hypoleuca*) [51]	4.9	13.1	0.18	0.29	144
Great tit (*Parus major*) [52]	6.7	25.3	0.45	0.18	88
Blue tit (*Cyanistes caeruleus*) [53]	13.2	50.6	0.69	0.23	104
House wren (*Troglodytes aedon*) [54] and ERAC unpublished	13.5	37.6	0.80	0.20	182
House martin (*Delichon urbica*) [55] and Jan Lifjeld, unpublished	13.8	47.4	0.84	0.22	56
Winter wren (*Troglodytes troglodytes*) [56]	16.3	37.9	0.74	0.26	29
Collared flycatcher (*Ficedula albicollis*) [57]	20.5	55.7	0.97	0.27	60
Bluethroat (*Luscinia svecica*) [58] and Arild Johnsen unpublished	23.6	45.2	0.69	0.44	261
Hooded warbler (*Wilsonia citrina*) [59,60]	27.1	35.9	0.48	0.68	117
Willow warbler (*Phylloscopus trochilus*) [61] and Jan Lifjeld, unpublished	33.1	56.3	0.93	0.49	71
Yellow warbler (*Setophaga petechia*) [62]	33.7	58.8	1.05	0.44	90
Black-throated blue warbler (*Setophaga caerulescens*) [63] and unpublished (Sara Kaiser, Scott Sillett, Mike Webster)	43.4	55.6	1.00	0.59	1287
Tree swallow (*Tachycineta bicolor*) [45]	47.9	82.1	2.00	0.40	67
Reed bunting (*Emberiza schoeniclus*) [56]	52.4	71.4	1.37	0.61	70

This simulation builds on ones recently used to explore bias in other analytical tools [22] and to understand how spatial constraints on extra-pair copulations affect the impact of extra-pair paternity on sexual selection [49]. They draw on an analytical framework developed by Brommer and colleagues [56,64]. Simulations and analyses were conducted in R 3.3.0 using *dplyr*, *tidyr*, and *ggplot2* [65,66,67,68]. For each of the conditions described below, 100 replicate populations were created for each species and condition set. 

**Figure 1:**
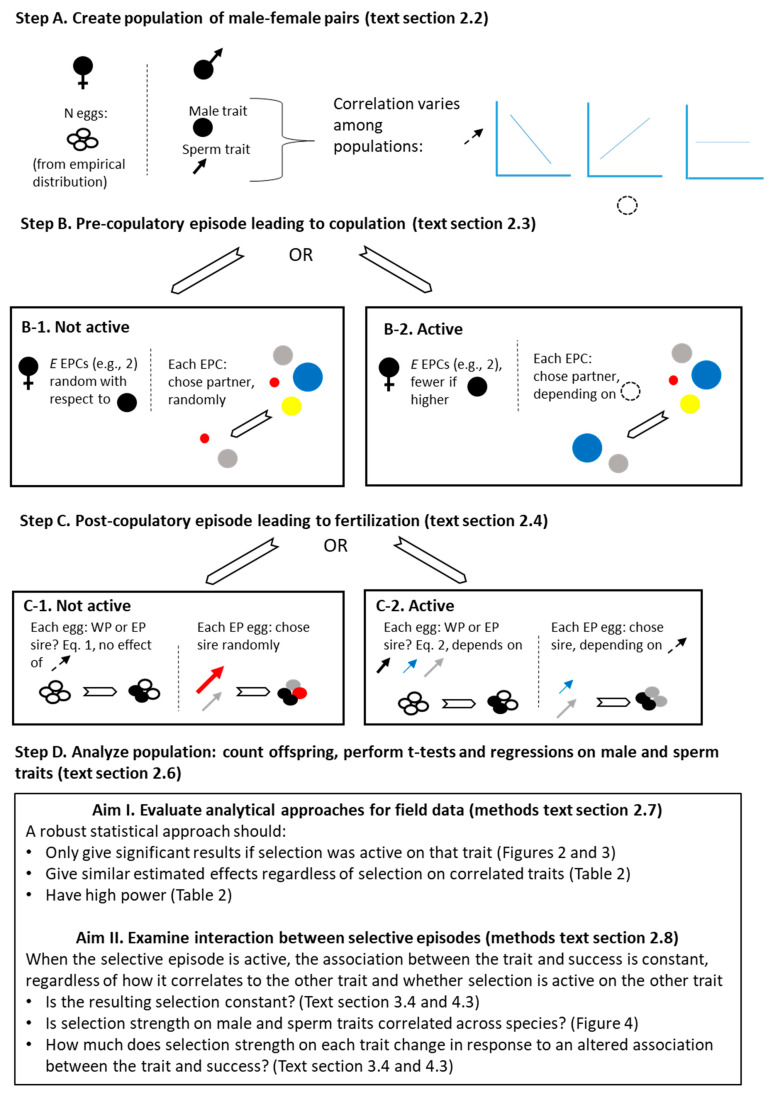
Schematic representation of simulation procedure and explanation of aims. Males (or the eggs they fertilize) are represented by unique colors. The male trait is indicated by a circle of varying size and the sperm trait by an arrow of varying length and width. The circle and arrow are dashed when they do not indicate specific individuals. Populations had either only pre-copulatory selection active (Steps B-2 and C-1), only post-copulatory selection active (Steps B-1 and C-2), or both episodes active (Steps B-2 and C-2). For each of these combinations, varying correlations between the male trait and sperm trait were tested. The female was assigned a number of extra-pair copulations (EPCs), *E*, by drawing from a Poisson distribution with species-specific mean *m; E* did (B-2) or did not (B-1) depend on the within-pair male’s male trait. From the available extra-pair males (four shown here, for illustration), *E* actual copulation partners were drawn, randomly (B-1) or depending on the extra-pair male’s male trait (B-2). The probability that each egg was fertilized by extra-pair sperm was calculated, either depending only on *E* and a species-specific term *s* (C-1, Equation (1)), or depending on *E*, *s*, and the relative sperm quality of the within-pair male and extra-pair copulation partners (C-2, Equation (2)). For extra-pair eggs, the sire was assigned from among the extra-pair copulation partners either randomly (C-1) or depending on their sperm trait (C-2). Illustrations of available sperm for population C-1 are from step B-1, and for C-2 from B-2. Aims are explained, with reference to methods sections and results text, figures and/or tables.

Simulations treated copulation as a separate, independent step from fertilization, detailed below (see also Figure 1). In all simulations, each male was assigned two traits, one representing a male secondary sexual trait and one representing a sperm trait. I refer to an episode of selection as “active” when the relevant trait impacted success (i.e., when the male trait impacted copulation success, pre-copulatory selection was active; when the sperm trait impacted fertilization success, post-copulatory selection was active). This phrasing attempts to distinguish the simulated process from its outcome (i.e., various measures or indicators of selection on the trait). Active selective episodes simulated directional selection on the traits; traits can therefore be thought of as quality traits representing “good genes” [69] or “good sperm” [70], as arbitrary attractive traits under runaway selection (including “sexy sperm”) [71,72,73,74,75,76], or as traits that make males or their sperm relatively successful in competition. In the main simulations, traits are associated with both within-pair and extra-pair success, i.e., losing fewer within-pair offspring to other males and gaining more extra-pair offspring in other nests, respectively. Additional simulation conditions where traits affected only extra-pair success are presented in the supplement.

### 2.2. Creating Individual Populations 

Each replicate population began with 400 male–female social pairs, on an evenly spaced 20 by 20 grid (where units are territory diameters). Males were assigned scores for the male and sperm traits randomly with respect to location. Scores were generated from multivariate normal distributions with mean of zero, standard deviation of 1, and a specified degree of correlation (values examined: −0.3, −0.2, −0.1, 0, 0.1, 0.2, and 0.3 in separate replicates; generated using package *MASS* [77]). Both trait distributions were shifted to have a minimum value of 0.1, as positive values were needed in downstream uses. Females were assigned clutch sizes by drawing with replacement from the empirical distribution of clutch sizes for the species; this was random with respect to the trait scores of her social partner and to territory location. 

### 2.3. Copulations 

To model copulations, I drew a list of 400 values from a Poisson distribution with a species-specific mean value *m.* Each value, *E*, represents the number of extra-pair copulations a female performs. As in [49], if pre-copulatory sexual selection was not active, values of *E* were assigned to females randomly. If pre-copulatory selection was active, to make males with higher male trait values less likely to lose paternity within their own broods, I sorted males in decreasing order of relative male trait value. I sorted the values of *E* in increasing order, then merged the two together according to order. Since extra-pair copulations were restricted to first- and second-degree neighbors, relative male trait scores were assessed by comparing the trait of the focal male to the mean traits of males whose territories were within 2.5 distance units. This process causes a strong correlation between the male trait and the number of extra-pair copulations performed by his social female (−0.65 < mean Pearson r per 100 populations < −0.93, across the 15 species); additional conditions where the male trait is uncorrelated with his social mate’s *E* are presented in the supplement. 

For each extra-pair copulation, a partner was identified. All first- and second-degree neighbors were available as partners, but closer males may be more likely to sire extra-pair offspring ([49] and references therein). To weight each male’s probability of being a copulation partner, I therefore divided the male trait score by the squared distance between his territory center and the female’s. For simulations where pre-copulatory selection was not active, I raised the male trait to the power of 0 before calculating the quotient, eliminating the impact of male traits [49]. Using this quotient as the weight, I drew with replacement the identity of the *E* extra-pair copulation partners for each female, using the *sample* function in R. This process introduces substantial stochasticity into the association between the male trait and copulation success, as a male’s probability of being drawn is proportional to his weighted trait score, relative to the sum of the weighted trait scores of all other available males. 

As a preliminary investigation into how selection on the male trait changed when the association between the trait and extra-pair copulation success was strengthened, I ran a further set of 100 populations per species. Here, the male trait was raised to the power of 9 (chosen arbitrarily; this was strongest selection modeled in [49]). I then used this new variable in place of the male trait in determining copulation patterns. For this pilot, only pre-copulatory selection was active, and the male and sperm traits were not correlated. 

### 2.4. Fertilizations 

For populations where post-copulatory selection was not active, I assigned fertilizations as in [22,49]. Specifically, fertilization was similar to a fair raffle, where all extra-pair copulation partners had an equal likelihood of achieving fertilization regardless of their sperm trait [78]. The relative probability that an extra-pair and a within-pair copulation results in fertilization depended on a species-specific term, *s*, representing the relative number of sperm inseminated by a single extra-pair copulation, compared to total number inseminated by the within-pair male [56,64]. All within-pair males are assumed to inseminate equal numbers of sperm. Specifically, the probability that each egg would be fertilized by an extra-pair sire, *f*, was given by

(1) $$f=\;\frac{E*s}{E*s+\left(1-s\right)}$$

where *E* is the number of extra-pair copulations performed by the female. After calculating *f* for each female, I determined if each egg in the clutch was fertilized by an extra-pair male by pulling from a Bernoulli distribution with mean *f*. I then assigned the sire for extra-pair fertilizations by drawing with replacement from the female’s list of extra-pair copulation partners, with no weighting. 

For populations where post-copulatory selection was active, I incorporated sperm trait scores in calculating *f*, and I used it in assigning the fathers of extra-pair offspring, approximating a loaded raffle [79]. Expanding on the notation from Equation (1), *f* was calculated as

(2) $$f=\;\frac{\sum_{i=1}^{E}s*EPS_{i}}{\sum_{i=1}^{E}s*EPS_{i}+\left(1-s\right)*WPS}$$

where *EPS_i_* is the sperm trait of the *i*-th extra-pair copulation partner, and *WPS* is the sperm trait of the within-pair male. Thus the probability that the egg is fertilized by an extra-pair male depends on the combined sperm traits of all the extra-pair copulation partners, relative to the sperm trait of the within-pair male. I determined whether an egg was sired by an extra-pair male by drawing from a Bernoulli distribution with mean *f.* I then assigned sires to the extra-pair eggs by drawing from the list of extra-pair copulation partners (with replacement), weighting by sperm trait, using the sample function in R. 

To assess how selection on sperm changes with a change in the association between the sperm trait and fertilization success, I ran an additional set of 100 populations per species. Here, analogously to the change in the association between the male trait and copulation success, I raised the sperm trait to the power of 9. I used this new variable in place of the sperm trait for calculating *f* (Equation (2)) and assigning extra-pair sires. Only post-copulatory selection was active, and male and sperm traits were not correlated.

This approach to simulating fertilizations makes a number of simplifications for convenience, detailed in the discussion (Section 4.4). The process does, however, result in a realistic distribution of extra-pair offspring among broods [22,49,56,64], and it allows controlled comparison across conditions. 

### 2.5. Estimating m and s

As described previously [22,49,64], the species-specific values of *m* and *s* were estimated using Bayesian analyses. Empirical data for each species on the number of extra-pair offspring and the total brood size were obtained from the literature and unpublished work (Table 1, [48]). As described above, a set of values of *E* can be drawn from the Poisson distribution, and these are used to calculate brood-specific values of *f* following Equation (1). The number of extra-pair offspring given a particular brood size can then be estimated using the probability mass function of the binomial distribution. Using a Bayesian approach, values of *m* and *s* are simultaneously optimized to best fit the empirical data. Values for the species used here were obtained from [64], or calculated using their code by [22,49] in *JAGS* [80], accessed through R using *rjags* [81] and *jagsUI* [82].

### 2.6. Measuring Selection within Each Replicate Population 

To avoid edge effects due to the spatial constraints on extra-pair copulations, I first tallied copulations and fertilizations across the entire population and then excluded males whose territories were within 3 distance units of the plot edge. Offspring in these edge broods, and copulations with these females, were thus included for focal males in the core study plot (*n* = 169 males). All tests were coded such that selection acting on the focal trait was expected to give a positive difference or parameter estimate. 

Following common practice for extra-pair paternity studies, I used unpaired t-tests to compare traits between males that did or did not lose paternity in their own brood (i.e., were or were not cuckolded). Additionally, using unpaired t-tests, I compared traits between males that did or did not sire extra-pair offspring in other broods. Pilot analyses using logistic regression with the proportion of offspring gave similar results but occasionally did not converge, complicating the automation of the simulations (not shown). In addition, I performed paired t-tests to compare traits of extra-pair males to the within-pair males they cuckolded. Paired t-tests have been criticized because the same individual male can appear multiple times within a dataset (i.e., by cuckolding or being cuckolded by multiple other males). Analogous methods using random slopes are possible [83], but are avoided here for simplicity. For these tests, only replicate populations with at least 3 males in each category were included. 

Because selection gradients are more directly related to evolutionary theory than are t-tests [84,85,86], I calculated univariate and multivariate selection gradients by regressing genetic offspring produced on male and sperm trait(s). I standardized predictor variables to have a mean of 0 and a variance of 1, and I standardized reproductive success by dividing by its population mean [87]. For univariate analyses, only one trait was used as the predictor in each model. This can represent species where the target trait of the non-focal episode of selection is not known. For the multivariate analysis, male and sperm traits were included in the same model as predictors of total offspring produced, as recommended for correlated variables [87]. 

Because pilot work showed that these analytical approaches performed poorly for sperm, I investigated three additional analyses for sperm traits only. First, I tested whether the sperm trait related to the number of females identified as extra-pair copulation partners (i.e., with shared offspring). To improve normality of residuals, I used the sperm trait as the response variable and number of females as the predictor. Second, I tested whether an extra-pair male’s sperm trait affected extra-pair fertilization success by regressing the proportion of extra-pair brood(s) sired on the sperm trait of extra-pair males. Only female–male combinations where the male had sired at least one extra-pair offspring were included, and the proportion of the brood was log-transformed for normality. Male identity was included as a random effect if any male was included more than once in the dataset (i.e., if he sired extra-pair offspring in more than one nest). Third, I tested whether a within-pair male’s sperm trait affected the proportion of his own brood that he sired, given that extra-pair copulation was known to have occurred. Here, I used proportion of within-pair offspring sired as the response variable and restricted the dataset to males that lost at least one within-pair offspring to extra-pair paternity. 

Many studies on the interaction between pre- and post-copulatory sexual selection partition variance in total reproductive success into pre- and post-copulatory components, rather than focusing on selection on traits [13,14,88,89,90]. To facilitate comparison with this work, I partitioned variance in reproductive success into components following [91] (see Supplementary Materials, Table S1). 

### 2.7. Evaluating Analytical Approaches (Aim I)

To examine statistical robustness, I evaluated whether the male trait vs. sperm trait correlation caused spurious significant results and bias in estimated effects. For tests with relatively low bias, I assessed relative statistical power. I assume that significant test results would be interpreted as evidence that the focal trait impacted success in its own episode of selection. Therefore, I consider significant results (in excess of expectations due to Type I error) for a trait when its selective episode was not active to be spurious. In addition, when the focal episode of selection was active, the trait was associated with success in a consistent manner. Therefore, an unbiased test should produce similar effects regardless of how the focal trait correlates with the other trait. 

To investigate spurious significant results, I examined populations where the focal episode of selection was not active and the non-focal episode was active. For each analytical approach, I constructed a separate linear mixed model to test whether the number of significant test results in the negative direction (response variable) depended on the male trait vs. sperm trait correlation (modeled as a categorical variable to improve normality of residuals), with species as a random effect. I examined only the negative-direction results to simplify analysis, since attempts to examine all significant results together produced strongly skewed residuals, and since visual inspection suggested that negative and positive direction results mirrored each other (see Results). 

To investigate bias in the estimated effects, I compared how estimated effects changed with the male trait vs. sperm trait correlation, depending on the active episodes of selection (focal only vs. both). Populations with only the focal episode active can be considered a reference where the estimated effects are expected to be independent of the male trait vs. sperm trait correlation, because selection on the other trait was not active (this expectation was supported; see Results). Finding a different pattern for populations where both episodes were active (i.e., a significant interaction between male trait vs. sperm trait correlation and active episodes) would therefore indicate bias caused by selection on the correlated trait. For each analytical approach, I constructed a separate linear mixed model with the estimated effect as the response variable (estimated effect for t-tests: difference between groups; for regression approaches: regression coefficient). The models included a random effect of species. The predictors in these models were the male trait vs. sperm trait correlation (modelled as a continuous variable), which episodes of selection were active, and an interaction between these two. To facilitate comparison across analytical approaches, I calculated an effect size (Cohen’s *d*) on the interaction term, following Equation (22) in [92] to account for repeated observations of the same species. Values of *d* approximately 0.2 are considered small, 0.5 are considered moderate, and 0.8 are considered large [92]. All mixed models used package *lme4* [93] and assessed significance using Sattherthwaite’s estimate of degrees of freedom, in package *lmerTest* [94].

Statistical bias could also cause spurious non-significant results, i.e., masking an underlying association between a trait and success, when traits are negatively correlated. To test this idea, for the analytical approaches showing substantial bias, I compared the number of non-significant (*p* > 0.05) test results (response variable) between populations where only the focal episode of selection was active and where both episodes of selection were active (predictor). I examined total non-significant results (in both the predicted and non-predicted directions), since bias could alter the apparent direction of the relationship. These tests examined only populations where the correlation between the male trait and sperm trait was −0.1, because stronger negative correlations between the male trait and sperm trait generated increasing numbers of significant results in the “wrong” direction. I constructed a separate linear mixed model for each test type, with species as a random effect. 

As an approximation of statistical power, I calculated the percent of significant results in the expected (positive) direction, for each unbiased analytical approach. 

### 2.8. Interaction between Episodes (Aim II)

I tested whether the strength of direct selection (i.e., the selection gradient from a multivariate selection analysis [87]) differed when only the focal episode or both episodes of selection were active, using the same mixed models as for testing bias in estimated effects. Here, rather than focus on the interaction term, the comparison of interest was whether selection differed when only one episode or both were active. 

I assessed whether direct selection on the two traits (calculated as the mean multivariate selection gradient from populations where both episodes of selection were active) was correlated across species using a generalized least squares (gls) model in *nlme* [95], correcting for phylogeny by specifying a correlation structure following Pagel’s lambda [96]. The phylogenetic structure was determined as the consensus tree calculated from 1000 trees downloaded from birdtree.org (Hackett backbone), using *phytools* [97]. To explore how simulation parameters affected the strength of selection, I constructed a phylogenetically controlled gls model with the average strength of direct selection in each species as the response variable and the simulation parameters *m* and *s* as predictors. To evaluate how much selection changed due to altering the association between a trait and success, I constructed a phylogenetically controlled gls model for each trait, with the strength of direct selection as the response variable and the association type (main vs. strengthened) as the predictor variable. Accounting for phylogeny did not substantively affect the results on statistical robustness (using packages *ape* [96] and *MCMCglmm* [98]; see details in Supplementary Materials). 

## 3. Results

### 3.1. General

The proportion of extra-pair offspring and the proportion of broods with at least one extra-pair offspring was slightly but significantly lower when only the pre-copulatory episode was active, compared to when only the post-copulatory episode or both episodes were active (t_31480_ > 11.1, *p* < 0.001; Cohen’s *d* for difference in extra-pair offspring 0.02, Figure S1; for extra-pair broods, *d* = 0.03). This is likely due to slight differences in the average value of *f* (the probability of an egg being fertilized by an extra-pair male) calculated via Equations (1) and (2). The proportion of extra-pair offspring and broods did not vary depending on the male trait vs. sperm quality correlation for simulations with a single episode of selection active (t_31480_ < 0.90, *p* > 0.3), but they were positively associated with the male trait vs. sperm quality correlation when both episodes were active (t_31480_ > 13.9, *p* < 0.001; Cohen’s *d* for the interaction for extra-pair young, 0.04, extra-pair broods, 0.02). Comparisons of selection would have been more robust if extra-pair paternity patterns were indistinguishable across treatments, but the scale of the variation observed was relatively minor, particularly in the context of substantial annual variation in extra-pair paternity rates within populations (e.g., [99], Figure S1). 

### 3.2. Evaluating Analytical Approaches (Aim I)

**Figure 2:**
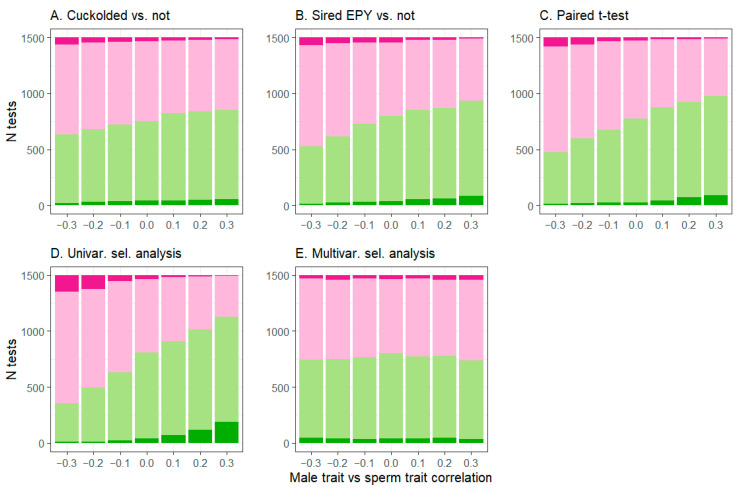
Spurious significant results for male traits (i.e., detected selection in individual populations where the male trait did not affect copulation success) were relatively uncommon for most analytical approaches. Pink colors indicate estimated negative selection and green colors indicate positive selection; intense colors are significant test results, while lighter colors are non-significant. Analytical approaches were (**A**): unpaired t-tests of males that lost paternity within their own broods (were cuckolded) or did not; (**B**): unpaired t-tests of males that gained extra-pair young (EPY) in other broods or did not; (**C**): paired comparisons of extra-pair sires to the within-pair males that they cuckolded; (**D**): univariate selection analysis regressing offspring sired on the male trait; and (**E**): multivariate selection analysis regressing offspring sired on both traits (with test results shown for the male trait only). Type 1 error is expected to produce only 5% (75) significant tests per column.

**Figure 3:**
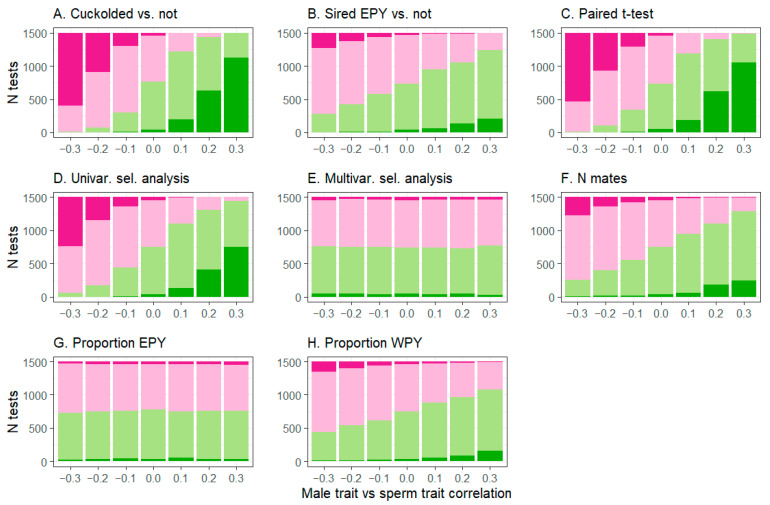
Spurious significant results for sperm traits (i.e., detected selection in individual populations where the sperm trait did not affect fertilization success) were common for most analytical approaches when the sperm trait correlated to a male trait under selection. Pink colors indicate estimated negative selection and green colors indicate positive selection; intense colors are significant test results, while lighter colors are non-significant. Analytical approaches were (**A**): unpaired t-tests of males that lost paternity within their own broods (were cuckolded) or did not; (**B**): unpaired t-tests of males that gained extra-pair young (EPY) in other broods or did not; (**C**): paired comparisons of extra-pair sires to the within-pair males that they cuckolded; (**D**): univariate selection analysis regressing offspring sired on the sperm trait; (**E**): multivariate selection analysis regressing offspring sired on both traits (with test results shown for the sperm trait only); (**F**) regression of the sperm trait on the number of detected extra-pair copulation partners; (**G**) regression of proportion of extra-pair brood sired on the sperm trait of extra-pair males, including only broods where the male sired at least one extra-pair young; and (**H**): regression of the proportion of within-pair young (WPY) on the sperm trait of the within-pair male, including only broods where the male lost paternity. Type 1 error is expected to produce only 5% (75) significant tests per column.

In populations where the focal episode of selection was not active and the male trait vs. sperm trait correlation was 0, approximately 2.5% of tests from individual populations were significant in the negative direction, as can be expected due to Type I error with α = 0.05 (Figure 2 and Figure 3, Table S2). However, as the absolute value of the male trait vs. sperm trait correlation increased, spurious significant test results became more common, for all analytical approaches except the multivariate selection analysis and the regression of proportion of extra-pair brood(s) sired on the sperm trait of extra-pair males (Figure 2 and Figure 3, Table 2, Table S2). Spurious results were quite frequent for some analyses on the sperm trait (Figure 3). The effect sizes of these spurious significant results were substantial for t-tests, where the difference between groups (mean ± SD, min–max) was 0.44 ± 0.14, 0.17–1.32, in units of trait standard deviations. It was also substantial for the regression of the sperm trait on the number of detected extra-pair copulation partners, where each additional copulation partner corresponded to a change of 0.25 ± 0.09, 0.11–0.72 standard deviations in the sperm trait. For other regression-based approaches, the effect sizes of spurious significant tests were lower (|mean estimated regression coefficient| <0.13). 

Substantial bias occurred in the estimated effects for many analytical approaches, particularly for the sperm trait (Table 2 and Table S3). As expected, when only the focal episode of selection was active, the estimated effects for the focal trait did not depend on the male trait vs. sperm trait correlation (|t_20982_| < 1.4, *p* > 0.15; see full results Table S3). However, the effect of the male trait vs. sperm trait correlation differed when both episodes were active, for all tests except the regression of proportion of extra-pair brood sired on the sperm trait of extra-pair males (Table 2 and Table S3, most tests: F_1,20982_ > 12, *p* < 0.001; the latter regression, F_1,20982_ = 2.83, *p* = 0.09). For male traits, the degree of bias was generally small (Cohen’s *d* < 0.2, Table 2), but for several analytical approaches for sperm traits, bias was extreme (Cohen’s *d* > 1, Table 2). For sperm traits, bias was low (|*d|* < 0.05) only for the multivariate selection analysis and the regression of proportion of extra-pair brood sired on the sperm trait of extra-pair males. Multivariate selection analysis is not expected to show bias due to correlated traits [87], and the significant (though very weak) pattern found here may actually reflect differences in the strength of selection due to differences in the frequency of extra-pair paternity across simulation conditions (see Section 3.1). Supporting this argument, effect sizes for multivariate selection analysis and frequency of extra-pair paternity are similar (all *d* ≤ 0.04), and variation among species in the two patterns are similar (Figures S1 and S2). 

The biased analytical approaches also showed spurious non-significant results, whereby selection on the sperm trait was less likely to be significant when the pre-copulatory episode was also active, and when the male trait vs. sperm correlation was weakly negative (−0.1; % fewer significant results ± SE: univariate selection gradient, 22.53 ± 3.2; paired t-test, 16.1 ± 2.4; unpaired t-tests, regression on detected extra-pair copulation partners, and regression on the proportion of within-pair young sired, approximately 6%; all F > 8.8, *p* < 0.01). 

Relative statistical power was high for male traits for most analytical approaches, with 99–100% of tests producing significant results in the expected direction, except for the comparison of males that did or did not sire extra-pair young (approximately 80% of tests, Table 2). Relative power for the unbiased analytical approaches for sperm was lower (50–60% of tests, Table 2). The high proportion of significant tests likely results from the large sample size (*n* = 169 males per population), the absence of noise due to randomly distributed nest failures, and the relatively strong associations modeled between male traits and copulation success. 

### 3.3. Evaluating Analytical Approaches: Additional Conditions (Aim I)

As in the main results, two analytical approaches (multivariate selection analysis and the regression of proportion of extra-pair brood sired on the sperm trait of extra-pair males) showed minimal bias in the additional simulation conditions presented in the supplement (Tables S2–S4). For other analytical approaches, the degree of bias differed qualitatively from the main results only as follows. When the male trait only affected success at gaining extra-pair copulations (and not within-pair success), two analyses on sperm became unbiased: the t-test comparing sperm of males that were or were not cuckolded, and the regression of proportion of within-pair young sired on the within-pair sperm trait. Bias in the univariate selection gradient on sperm was somewhat reduced, but still moderate (Cohen’s *d* 0.37). In this condition, bias increased to a moderate level (Cohen’s *d* > 0.2) for two analyses on the male trait: the paired t-test of extra-pair males and the within-pair males they cuckolded, and the univariate selection gradient. When the sperm trait affected only success at fertilizing extra-pair eggs (and not within-pair success), bias was reduced for all analytical approaches for the male trait. Bias was generally increased in populations where the association between the non-focal trait and success were stronger. 

### 3.4. Interaction between Episodes (Aim II)

Direct selection on the male trait (i.e., the selection gradient from multivariate selection analysis) was marginally higher when both episodes of selection were active than when only the focal episode was active (estimated difference with male trait vs. sperm trait correlation = 0: t_20980_ = 4.42, *p* < 0.001; Cohen’s *d:* 0.02). Direct selection on the sperm trait did not differ when both episodes or only the focal episode was active (t_20980_ = −1.59, *p* = 0.11). For both traits, selection increased slightly with increasing male trait vs. sperm trait correlation, when both episodes were active (t_20980_ > 3.7, *p* < 0.001, Cohen’s *d* 0.02). These slight differences appear to be driven by variation in the proportion of extra-pair offspring across conditions (see also Section 3.1 and Section 3.2). That is, effect sizes for selection on the male trait and for the frequency of extra-pair offspring were similar, and within-species patterns were similar (compare Figures S1 and S2). Notably, these effect sizes were very low, indicating that the difference is unlikely to be biologically important. In contrast to direct selection, opportunity for selection increased with increasing male trait vs. sperm trait correlations to a greater extent, unlikely to be fully explained by variation in the frequency of extra-pair offspring, for most fitness components (Supplementary Materials, Table S5, Figure S3). 

**Figure 4:**
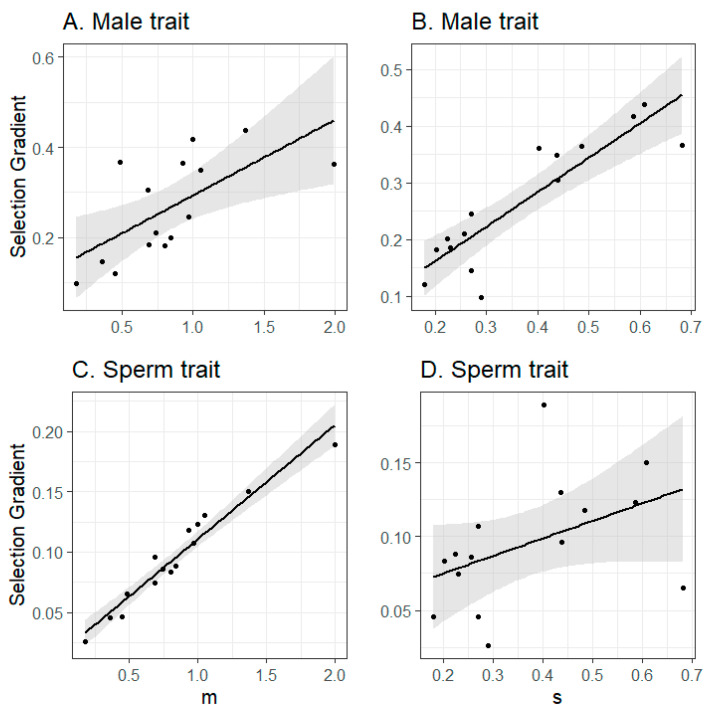
The strength of direct selection on male traits (top row (**A**,**B**)) or sperm traits (bottom row (**C**,**D**)) depending on the species’ value of *m* (left column; Poisson-distributed mean number of extra-pair copulation partners) and *s* (right column; variable affecting the likelihood that an extra-pair copulation results in fertilization). Shaded areas represent 95% confidence interval. Direct selection strength was estimated as the mean value from the multivariate selection analysis from populations where both traits were under selection, across all levels of male trait vs. sperm trait correlation. Raw values are presented; statistical tests controlled for phylogenetic relatedness.

The strength of direct selection on male and sperm traits was positively correlated across species (t_13_ = 4.68, *p* < 0.001, estimated correlation, 0.80). Under the main simulation conditions, selection on male traits was higher than selection on sperm traits (model with selection on male traits as the response, selection on sperm traits as the predictor, intercept significantly greater than 0: 0.10 ± 0.04 (SE), t_13_ = 2.49, *p* = 0.03; and slope substantially greater than one, 1.63 ± 0.34). Controlling for phylogeny, selection depended on *m* (mean number of extra-pair copulations per female) and *s* (relating to the probability that extra-pair sperm achieve fertilization, Figure 4). Specifically, selection on male traits was strongly affected by *s* (t_12_ = 76.46, *p* < 0.001, partial r = 0.99) and *m* (t_12_ = 14.91, *p* < 0.001, partial r = 0.97). Selection on sperm traits was affected by *m* (t_12_ = 213.53, *p* < 0.001, partial r = 0.99) but not *s* (t_12_ = 0.14, *p* = 0.89, partial r = 0.04; Figure 4).

Selection on the male traits increased more dramatically than selection on sperm traits following analogous changes in how each trait was associated with success in its respective episode of selection (estimated difference ± SE at intercept for male trait: 0.26 ± 0.08, t_28_ = 3.23, *p* = 0.003, for sperm trait: 0.10 ± 0.02, t_28_ = 4.36, *p* > 0.001; estimated slope for male trait: 0.26 ± 0.05, t_28_ = 5.48, *p* < 0.001, for sperm trait: 0.05 ± 0.01, t_28_ = 4.54, *p* > 0.001). The average percent increase in selection on the male trait (mean ± SD) was 92.5 ± 32.2%, compared to 41.5 ± 25.3% for the sperm traits.

## 4. Discussion

### 4.1. Analytical Approaches (Aim I): Recommendations

Most commonly-applied analytical approaches produced misleading results for sperm for socially monogamous species with extra-pair paternity: they spuriously implied selection on sperm in populations where the sperm trait did not impact fertilization success; estimated effects were biased when both pre- and post-copulatory episodes of selection were active; and the impact of the sperm trait on fertilization success was obscured when both episodes were active and the male trait was weakly negatively correlated with the sperm trait. These analytical approaches should therefore be avoided, unless researchers can be certain that no correlated male traits are under selection. Previous interpretations (e.g., [40]) may therefore require re-evaluation. 

Two analytical approaches were robust for investigating selection on sperm traits. Multivariate selection analysis, where the number of offspring produced is simultaneously regressed on multiple traits, is the recommended approach for studying selection on correlated traits [87], and it performed well here. However, this approach is only possible when relevant correlated traits are known. If relevant correlated traits exist but are not included in the selection analysis, researchers are essentially conducting a univariate selection analysis. As shown here and previously recognized [87], failure to account for correlated traits under selection can cause substantial bias in selection gradients, and thus incorrect interpretations for the impact of sperm traits on fertilization success. To avoid such mis-interpretations, researchers should carefully consider whether the most relevant traits under pre-copulatory sexual selection are known, before deciding to use multivariate selection analysis. The other robust test does not require information on correlated male traits. That test regressed the proportion of extra-pair brood(s) sired on the sperm trait of extra-pair males, including only broods where the male sired at least one extra-pair young. This analytical approach should be feasible for standard field studies, regardless of traits under pre-copulatory sexual selection, and is therefore recommended. Since statistical power may be low, detailed descriptions of test results should be provided even for non-significant tests, to facilitate meta-analysis.

In contrast to the relative paucity of tests that were reliable for sperm, most tests gave only slightly biased results for the male trait (Cohen’s *d* < approximately 0.2), as long as male trait strongly affected within-pair success and the impact of sperm traits on fertilization success was not too strong (Table S4). 

### 4.2. Analytical Approaches (Aim I): Explaining Observed Patterns

Two factors explain why most analytical approaches more strongly reflect copulation patterns than fertilization patterns, resulting in robustness for male traits and bias for sperm traits. To most intuitively describe these factors, the discussion below frames the traits as negatively correlated quality traits, although the logic applies regardless of the direction of the correlation or the cause of the association between the trait and success. 

First, many females performed no (or few) extra-pair copulations, leading to complete (or high) success siring within-pair offspring, regardless of the within-pair male’s sperm quality. This factor is most relevant to the unpaired t-test comparing males that were or were not cuckolded within their own broods (since many males in the successful, un-cuckolded category would have low-quality sperm), for the univariate selection gradient (since males with low-quality sperm would produce substantial numbers of offspring due to their within-pair success only), and for the regression of proportion of within-pair young sired on within-pair male sperm (since females paired to males with low-quality sperm performed few extra-pair copulations). The simulated association between male quality and the number of extra-pair copulations performed by the social mate was quite high in the main results, resulting in the strong bias shown here. In supplementary simulation conditions where the male trait did not affect the number of extra-pair copulations his within-pair female performed, bias was eliminated for the t-test and the regression on the proportion of within-pair young, and reduced (though still substantial, likely due to the impact of male quality on obtaining extra-pair copulations) for the univariate selection gradient (Tables S3 and S4). For study systems where the number of extra-pair copulations performed by a female is independent of her social mate’s quality, the former two tests may therefore give unbiased results for sperm traits. However, they may still be unadvisable, as estimated effects were low (see reference category estimates, Table S4; and note that bias was increased for several tests on male traits in this condition). 

Second, low quality sperm was not precluded from fertilizing eggs, but rather fertilized them at lower levels than high-quality sperm. This factor is relevant for the unpaired t-test comparing males that did or did not sire extra-pair offspring in other broods: a male only needed to fertilize one egg with one extra-pair female to be considered successful. Successful extra-pair sires therefore represent a mix of males whose high male quality enabled them to copulate with one or more females (even if low sperm quality caused them to fertilize few eggs) and males whose high sperm quality increased their chances of fertilizing eggs following extra-pair copulations (although their low male quality caused them to obtain few extra-pair copulations). This mixture of phenotypes for “successful” males likely explains why this analytical approach is more biased for male traits than were other approaches. This factor similarly explains bias for the regression of extra-pair sperm quality on the number of detected mating partners. Males with more detected copulation partners represent males that copulated with more females (despite perhaps siring few eggs per copulation), as well as males whose sperm was more successful at fertilizing eggs following extra-pair copulation. The bias observed in the results of this test implies that copulation success has a greater impact on the results. 

For the paired comparison of extra-pair males to the within-pair males that they cuckolded, both of the above factors are relevant. If a male’s social partner did not perform any extra-pair copulations, he would not be included in such an analysis. Males that appear in this analysis as within-pair, cuckolded males are therefore more likely to be of low male quality and high sperm quality. The extra-pair copulation partner only needs to fertilize one egg to be included in the dataset, and this is possible even if he has low sperm quality, due to the stochastic assignment of fathers. When an extra-pair male with high male quality and low sperm quality copulates but does not fertilize any eggs, the result is simply a smaller sample size for the paired comparison.

These two factors also explain why the regression of proportion of extra-pair brood sired on extra-pair sperm quality was effective. Because the response variable is a continuous rather than a binary variable, more of the sperm-related variation in fertilization success is captured. The impact of variation in copulation success is reduced, because only males that sired at least one offspring with that female (i.e., that copulated with her at least once) were included in the dataset. Repeated copulations between the same individuals were not frequent for the main simulation conditions, and bias in this test increased in supplementary conditions with more-frequent repeated copulations, where copulation success had a stronger impact on the datasets. However, bias was still fairly low under the conditions simulated (Table S4, |*d*| < 0.09). This test thus appears likely to be unbiased across a wide range of socially monogamous species with extra-pair paternity, so long as fertilization success is independent of male quality. It may perform better in species with relatively large brood sizes, where more precise estimates of proportion are possible. A draw-back of this approach is that the proportion of an extra-pair brood sired depends not only on the male’s own sperm trait, but also on the sperm quality of the within-pair male and all the other extra-pair copulation partners. Competing males could represent a non-random subset of the population, for example if males in spatially clustered high-quality habitat have high quality sperm [100,101]. Such stochasticity in the competing males’ sperm quality may introduce substantial sampling variance to this analysis (as in other analyses: [102,103]), and may partly explain the low statistical power of this test. 

### 4.3. Interaction between Episodes in Socially Monogamous Species with Extra-Pair Paternity (Aim II)

These simulations did not support the hypothesis that pre- and post-copulatory selective episodes interact synergistically or in opposition [12,13]. Instead, direct selection on each trait (estimated as the multivariate selection gradient) was similar whether or not the selective episode for the other trait was active (i.e., whether or not the other trait impacted copulation or fertilization success). These results do not directly contradict the hypothesis of synergy, but rather highlight the need for precise wording in framing that hypothesis. Simulation results differed for direct selection and opportunity for selection, which is the metric most commonly used in studying this synergy hypothesis (reviewed in [13]). As described in the supplement, opportunity for selection increased as expected when both episodes of selection were active and success was positively correlated across episodes. The difference in results for direct selection on the traits and opportunity for selection illustrates that these metrics are not equivalent, as is already recognized [104]. A recently-introduced modified approach to calculating opportunity for selection may help to reconcile these two approaches, and should be investigated further [105]. Other studies focus on total selection on the trait (i.e, combined direct and indirect selection, e.g., [90]), similar to the univariate selection gradient measured here. The univariate selection gradient increased when both episodes of selection were active and when the traits were positively correlated. This pattern was here viewed as bias, under the assumption that test results would be interpreted as evidence that the traits impact success in their own selective episodes. From the perspective of total selection experienced, the univariate selection gradients here are not biased. However, distinguishing between direct and indirect selection has important implications for how traits will evolve [87], as does understanding whether traits are correlated phenotypically or genetically [106]. Notably, this simulation explicitly caused fertilization success to be affected only by the sperm trait, while in some species sperm use by the female can also depend on the male’s phenotype [88,107]. In such cases, synergistic effects on direct selection on the male trait may be expected. 

While selection on male and sperm traits were independent within species, they were positively correlated across species, and I suggest that this pattern will be common in socially monogamous species with extra-pair paternity. Assuming that extra-pair paternity rather than social pairing success drives sexual selection on male traits in this mating system [91,108,109], and assuming that within-pair partners copulate, the behavior (extra-pair copulation) that causes pre-copulatory sexual selection necessarily also creates the conditions for post-copulatory sexual selection. This inherent connection between pre- and post-copulatory sexual selection was apparent in the simulation results, in that the strength of selection on both traits increased with increasing frequency of extra-pair copulations (*m*). Selection on the male trait was more tightly associated with *s* than *m*, while selection on the sperm trait was only associated with *m.* The latter result agrees with comparative evidence from other mating systems. When females have a relatively low probability of copulating with multiple males (here, low *m*; in other studies, due to males controlling copulatory access [17], or due to low population density [8]), males show reduced investment into post-copulatory phenotypes, consistent with the lower strength of selection observed here. The overall positive correlation between selection strength on male and sperm traits is corroborated by comparative studies in birds that find positive associations between pre- and post-copulatory traits [110,111]. In other mating systems, where pre- and post-copulatory sexual selection are not necessarily linked (for example, in a lekking species where males may vary substantially in copulation success and may not face sperm competition, if females copulate only once), the strength of selection on male and sperm traits may be uncorrelated or negatively correlated (see also [9]).

Although selection on male and sperm traits were correlated across species, selection on sperm traits was considerably weaker than selection on male traits, under the main simulation conditions. These results are best viewed as tentative, since the strength of selection depended on how the simulation associated the trait with success, and, indeed, selection strength on the male and sperm trait was similar in supplementary conditions where the male trait did not affect the number of extra-pair copulations his social partner performed (Table S3). However, analogous alterations to the association between the traits and extra-pair copulation or fertilization success produced approximately twice as much increase in selection strength for male traits, compared to sperm traits. Thus, the scope for selection on sperm may be more inherently limited than the scope for selection on male traits in socially monogamous species with extra-pair paternity, as intuited previously for other mating systems [14,15,16], but see [105]. 

### 4.4. Simplifying Assumptions in the Simulation 

This simulation simplified fertilization as follows. Fertilization success did not depend on the order or timing of copulations [112,113], and it depended only on sperm quality, not on the phenotype of the male himself [88,107,114]. Sperm traits were constant for each individual, excluding sperm depletion due to repeated copulations [115] and strategic sperm allocation depending on the female’s mating history [78,116] or the male’s likelihood of obtaining further copulations [117]. Because only a single breeding season was simulated, I examined only annual, not lifetime, reproductive success, and age-related changes in sperm did not occur [118]. Embryos did not die due to excessive polyspermy, which is thought to be a risk for females copulating with multiple males with highly competitive sperm [119,120] (although some degree of polyspermy may be beneficial in birds [121,122]). The same sperm trait was positively associated both with a “defensive” role in fertilizing within-pair eggs and an “offensive” role in fertilizing extra-pair eggs [46,123], and a single sperm trait drove fertilization success [124]. All of these complexities are likely to weaken or obscure the relationship between sperm traits and fertilization success, because variation in fertilization success is explained by the other factors [14,88,89] or because selection on the sperm trait is non-linear. Thus including these additional factors in simulations seems most likely to weaken the observed selection on sperm traits, without removing the biases observed. 

Cryptic female choice mechanisms were also substantially simplified, in part because mechanistic knowledge in birds is limited. Female propensity to copulate again did not depend on ejaculate contents from previous copulations (as in *Drosophila* [125]). Additionally, females did not alter investment into eggs or offspring care, or (relevant for viviparous animals) selectively abort fetuses depending on the paternity of the embryo [5]. Such processes could result in increased bias in analyses of pre-copulatory selection, compared to results here, because they alter the mechanisms linking male phenotype to copulation success, or linking copulation to fertilizing robust, viable eggs. 

Simulations assumed all males were fertile. If substantial proportions of males are infertile (as occurs surprising often, [126]), and infertility relates to the measured sperm trait, biases in these statistical tests may be reduced because infertile males would have low or no reproductive success regardless of their copulation success. 

Finally, extra-pair copulations were assumed to follow a Poisson distribution with mean *m*, while an overdispersed Poisson distribution may be more realistic. Because the model parameters *m* and *s* (the sperm competition parameter) were simultaneously inferred, changing the assumed distribution of *m* would likely change the estimated value of *s.* For example, under an overdispersed Poisson model, where females on average perform more extra-pair copulations, the estimated value of *s* would need to be lower in order to achieve a realistic distribution of extra-pair offspring (see also [22]). How such a change would impact the statistical biases presented here is unclear. However, it seems most likely that for species with small to moderate clutch sizes, an increased proportion of extra-pair copulations would result in no fertilizations [22,127]. This in turn may result in increased noise for analyses on male traits and perhaps increased signal for analyses on sperm traits. 

### 4.5. Limitations for Interpretation

These simulations assumed that directional pre- and post-copulatory selection occurred, but they cannot provide insight into why such selection occurs. Extra-pair paternity has long been thought to be driven by female choice for “good genes”, whereby females obtain a fitness benefit by copulating with extra-pair males of superior additive genetic quality compared to their within-pair mate (though across species evidence for this hypothesis is limited [83,120,128]; see also [33,129]). It is unclear whether female preferences for both male and sperm traits should evolve via good genes mechanisms in the same population. Females are expected to use the single most reliable male quality signal, rather than use multiple male quality signals, according to theoretical work on handicap signals [130,131] (but see review in [132]). Whether that result would generalize to traits across separate episodes of selection is unclear. However, theoretical work on speciation, explicitly examining both episodes of selection, finds a similar result. Lorch and Servedio [133] used a genetically explicit model of hybridization, where hybrid offspring have low fitness, to explore selection on conspecific mate and sperm choice. They find that an adaptive preference for either the conspecific male phenotype or the conspecific sperm phenotype can evolve, but that the presence of one barrier inhibits the evolution of the other. The inhibition presumably occurs because the preferences are redundant, such that if one has evolved, there is little selective pressure to drive the evolution of the other [133]. If we view this model as being analogous to a strong good genes effect, it may indicate that selection on both sperm and male traits are unlikely to be maintained over time by a good genes mechanism. 

These simulations also assumed a correlation between the male and sperm traits. This approach differs from most related models, which often draw on game theory approaches to predict how males can optimally allocate finite resources between pre- and post-copulatory traits [6,18]. Those studies seek to understand what male trait vs. sperm trait correlation is selectively advantageous. Here, with a complementary approach, I ask how selection acts on each trait, given a particular level of correlation. 

## 5. Conclusions

The difficulty of directly observing copulations and fertilizations for most species results in many studies inferring success from parentage data. However, inferring copulation and fertilization success from parentage data creates problems in understanding sexual selection, as suggested in several previous studies [22,47,134,135] and in the current results. This practice can produce grossly misleading results for selection on sperm in socially monogamous species with extra-pair paternity, depending on the analytical approaches applied. Reliable methods for understanding how sperm traits affect fertilization success must either control for correlated traits involved in copulation success (i.e., performing multivariate selection analysis), or, if such traits are not known, regressing the proportion of extra-pair broods sired on the sperm trait of extra-pair males, considering only male–female sets that produced extra-pair offspring together. Using these unbiased analytical approaches can allow for more meaningful study of the interaction between pre- and post-copulatory sexual selection, even if copulation and fertilization success cannot be directly measured. For socially monogamous species with extra-pair paternity, selection on male and sperm traits may be generally positively correlated across species, and selection on the male traits appears likely to be stronger than selection on sperm traits. 

## Figures and Tables

**Table 2:** Assessment of bias and relative statistical power for several analytical approaches for detecting selection on male and sperm traits. Spurious significance shows how, in individual populations where the trait is not impacting success, the male trait vs. sperm trait correlation affects the number of significant results (in the negative direction). The bias in estimated effects reflects how much the estimated effect (difference between groups or regression coefficient) changed due to selection on a correlated trait. Specifically, it gives the Cohen’s *d* effect size of the interaction between the male trait vs. sperm trait correlation and a variable identifying whether only the focal or both selective episodes were active. Relative power is approximated by the percent of significant results in the expected direction for populations with only the focal trait under selection or with both traits under selection. This is provided only for tests without substantial bias.

Analytical Approach	Focal Trait	Spurious Significance	Bias in Estimated Effects (Cohen’s *d*)	Power (% Significant)
Unpaired *t*-test: cuckolded vs. not cuckolded within-pair males				
	Male trait	F_6,98_ = 6.06, *p* < 0.001	0.08	100, 100
	Sperm trait	F_6,84_ = 419.87, *p* < 0.001	1.61	not tested
Unpaired t-test: males that sired vs. did not sire extra-pair offspring in other broods				
	Male trait	F_6,84_ = 8.11, *p* < 0.001	0.18	81.8, 80.6
	Sperm trait	F_8,84_ = 62.71, *p* < 0.001	0.49	not tested
Paired *t*-test: extra-pair males and the within-pair males they cuckolded				
	Male trait	F_6,98_ = 17.70, *p* < 0.001	0.10	100, 100
	Sperm trait	F_6,84_ = 387.96, *p* < 0.001	1.28	not tested
Univariate selection gradient: offspring produced regressed on one trait				
	Male trait	F_6,84_ = 38.73, *p* < 0.001	0.18	99.4, 99.3
	Sperm trait	F_6,84_ = 105.37, *p* < 0.001	0.88	not tested
Multivariate selection gradient: offspring produced regressed on both traits				
	Male trait	F_6,84_ = 1.00, *p* = 0.43	0.02	99.3, 99.3
	Sperm trait	F_6,98_ = 2.05, *p* = 0.07	0.03	62.6, 66.4
Regression of trait on detected extra-pair copulation partners				
	Sperm trait	F_6,84_ = 59.92, *p* < 0.001	0.47	not tested
Regression of proportion of extra-pair brood sired on sperm trait of extra-pair males, among broods where that male sired ≥ 1 extra-pair young				
	Sperm trait	F_6,98_ = 0.86. *p* = 0.53	−0.02	52.4, 52.3
Regression of proportion of within-pair offspring sired on the sperm trait of the within-pair male, among broods with ≥ 1 extra-pair young				
	Sperm trait	F_6,84_ = 19.29, *p* < 0.001	0.43	not tested

## Data Availability

Code needed to perform simulations is included as a Supplementary file (Simulation Code.R). Raw data used in the simulations are openly available in Dryad at https://datadryad.org/stash/dataset/doi:10.5061/dryad.8kprr4xjk (accessed on 10 March 2021) reference [48].

## Supplementary Materials

The following are available online at https://www.mdpi.com/2073-4409/10/3/620/s1 Supplemental text describing methods, and R code for simulating populations. Figure S1. Proportion of extra-pair young depending on active selective episodes and male trait vs. sperm trait correlation. Figure S2. Strength of selection on the male trait when both episodes of selection were active or only pre-copulatory episode was active. Figure S3. Opportunity for selection and its components, as a function of the male trait vs. sperm trait correlation and which episodes of selection were active. Table S1: Partitioning total variance in reproductive success, *T*, into copulation success, *C*, average fecundity of the female mates, *N*, and fertilization success, *F*. Table S2: Complete model results for the number of spurious significant test results in the negative direction, for the main models and for additional simulation conditions presented only in the supplement. Table S3: Full model results for estimated effects of different analytical approaches, for the main results presented in the text and for additional simulation conditions. Table S4. Estimated effect size (Cohen’s *d*) for bias in parameter estimates due to correlated traits for additional simulation conditions described in the supplement. Table S5. Model results for how components of opportunity for selection change depending on which episodes of selection were active, the male trait vs. sperm trait correlation, and their interaction.
