# Supplementary material for: Measuring Pre- and Post-Copulatory Sexual Selection and Their Interaction in Socially Monogamous Species with Extra-Pair Paternity
Source: Cells. 2021 Mar 11;10(3):620. doi: 10.3390/cells10030620 (PMC7999480; doi:10.3390/cells10030620)
Supplement: Supplementary file 1 [file cells-10-00620-s001.zip › supplementary materials/cells-1056698 sm 3.10.pdf]

Supplementary material

1. Additional Simulation Conditions: Methods

To assess the robustness of the results to different assumptions about extra-pair copulation and fertilization processes, I made four modifications to the basic simulation. (1) To dissociate within-pair and extra-pair copulation success, I assigned the value of E randomly with respect to the within-pair male trait, but caused the male trait to correlate with extra-pair copulation success as in the main simulations. (2) I removed spatial constraints on extra-pair copulations. In determining a female's value of E, I arranged all males in decreasing order of the trait value (rather than relative trait value); any male was a potential extra-pair copulation partner for any female; and a male's likelihood of copulating with a female depended only on his trait score, rather than on the quotient of trait and distance squared. (3) I dissociated within-pair and extra-pair fertilization success. Here, I used Equation 1 from the main paper to calculate f, rather than Equation 2, but I assigned extra-pair sires to extra-pair eggs as in the main simulation. (4) I strengthened the impact of the non-focal trait on success; this was particularly relevant as a way to increase the frequency of repeated copulations between the same extra-pair male and female [1], as a potential additional source of bias for analyses on sperm. To increase the impact of the male trait, I raised the male trait to the power of 9 [1], and used this variable, rather than the male trait itself, for determining E and for assigning extra-pair copulation partners. To increase the impact of the sperm trait, I raised the sperm trait to the power of 9, and used this variable rather than the sperm trait to calculate f and to assign extra-pair fertilization.

For these conditions, I simulated 100 replicate populations per species, with male–sperm trait correlations of −0.3 and 0.3 only, to reduce computational burden. Since the male–sperm train correlation appeared linearly related in the main simulation results (not shown), this simplification should not impact interpretations substantially. Note that additional populations, similar to conditions 4 above, were run where the male and sperm trait were not correlated (see main text).

2. Phylogenetically Controlled Analyses

To conduct phylogenetically controlled versions of the mixed models on statistical bias, presented in the main text, I downloaded 1000 trees from the Hackett backbone from birdtrees.org, then calculated the consensus tree of these replicates using *phyltools* [2]. I then included this consensus phylogeny, as well as a random effect of species identity, in generalized linear mixed models using *MCMCglmm* [3]. Fixed effects and data subsets were the same as described in the main text. I constructed models to test how the number of significant results in the negative direction changed with male–sperm trait correlation when the focal episode of selection was not active (directly comparable to the first set of tests for Aim I; with the male–sperm trait correlation as a categorical variable). For comparing estimated effects (second set of tests for Aim I; first tests for Aim II), I reduced computational burden by first calculating the average effect (difference between groups, or regression coefficient) across the 100 replicate populations, for each species and set of simulation conditions. I then used these averages to test for the interaction between active episodes and male–sperm trait correlation (as a continuous variable; Aim I) and to test whether the activation of the non-focal episode impacted selection in the focal episode (Aim II; multivariate selection gradients only). For all MCMCglmm models, I ran 2,000,000 iterations, with a burn-in of 1000 and thinning = 500. Priors were set with V = 1 and nu = 0.02 for the random effects of species and phylogeny, as well as for residual variance, as recommended in [4]. Convergence was assessed by visual inspection of plots, as well as the Heidelberger and Welch's convergence diagnostic. This diagnostic performs poorly for parameters with a mean of 0, and indeed, gave a "failed" score for the effect of the male–sperm trait correlation in predicting estimated test effects (expected to be, and estimated close to, 0 in the reference category). If other fixed effects did not "pass" this diagnostic, I ran 5,000,000 iterations of the model. Only

one other parameter estimate failed this test after running 5,000,000 iterations; it was also estimated at close to 0, and therefore I concluded that the models had converged sufficiently. This package produces pairwise comparisons between the reference level and other levels, rather than overall F-tests, which somewhat complicated comparison to the main results.

For the number of spurious significant results, all analytical approaches that resulted in a significant F-test in Table 2 showed a similar pattern in the MCMCglmm results. Specifically, the number of significant results was significantly higher for the male–sperm trait correlation of −0.3 (compared to the reference correlation of 0), and the number of spurious significant results decreased with less-negative male–sperm trait correlations (consistent with the pattern in Fig. 2,3; full results not shown). For the multivariate regression on male traits and the regression of the proportion of extra-pair brood sired on the sperm trait of the extra-pair male, no categories differed from the reference value (full results not shown), consistent with the non-significant F tests (Table 2). For the multivariate selection gradient on the sperm trait, the number of spurious results differed for the male–sperm trait correlation of −0.2 and 0.1, but there was no overall pattern of increasing or decreasing number of spurious results, nor were estimated effects large (with these categories showing 1.7 and 1 fewer spurious significant result, respectively, than the reference; full results not shown). I therefore interpret this result as not substantively different from the main results.

For the size of estimated effects, I considered results from the MCMCglmm analysis completely consistent with main results if the estimated interaction term was less than 10% different from those for the main results (in Table S3), for main tests with Cohen's d > 0.08. For the main results with smaller Cohen's d, MCMCglmm resulted in non-significant interaction terms, which I interpret as consistent. Among tests with Cohen's d > 0.08, results were consistent except as follows. MCMCglmm indicated a significant interaction term, but with smaller estimated effects, for t-tests on sperm quality of males that were or were not cuckolded (estimated effect was 66% of that for main results); t-tests on sperm traits between males that did or did not sire extra-pair offspring in other nests (45%); paired t-tests of sperm quality (75%); and the regression of the sperm trait on detected extra-pair copulation partners (50%; full results not shown). I consider these substantively similar.

For Aim II, the MCMCglmm results indicated that selection in one episode did not depend on whether the other episode was active (full results not shown).

3. Partitioning Variance in Male Reproductive Success (Opportunity for selection): Methods

For comparability with other studies (reviewed in [5]), I partitioned variance in total reproductive success following [6]. Total reproductive success (i.e., number of offspring sired) was partitioned into two additive components, within-pair and extra-pair success, and each of these components was further partitioned into three multiplicative components representing copulation success, fecundity of the female copulation partner(s), and fertilization success [6]. Copulation success was the number of copulation partners a male had, regardless of whether he fertilized any eggs with her and regardless of number of copulations together. Within-pair copulation success was 1 for all males. Fecundity was the average clutch size of all extra-pair copulation partners, or the clutch size of the within-pair female. Fertilization success was the proportion of eggs fertilized (for extra-pair copulations, calculated as total number of eggs fertilized, divided by the sum of the clutch sizes of all extra-pair copulation partners). To avoid inflating variance terms, males that did not obtain extra-pair copulations were excluded from calculating terms that included extra-pair mate fecundity and fertilization success of extra-pair eggs [7]. Covariance terms between all pairs were also calculated (Table S1). Each variance or covariance component was scaled by multiplying by the appropriate squared mean or mean terms [6](Table S1). In order to discuss these variance terms as opportunity for selection and to facilitate comparison across species, each term was then divided by the square mean overall reproductive success [6].

To examine whether opportunity for selection in one episode depended on whether the other episode was active, I created a separate linear mixed model for each of the components of opportunity for selection, except for those involving within-pair copulation success (which was invariant in the simulation, resulting in values of 0 for all terms). The component of opportunity for selection was the response variable and species was a random effect. Fixed effects were the male–sperm quality correlation (treated as a continuous variable), a variable indicating which episodes of selection were active, and the interaction between these two. The interaction was removed if it was non-significant ($p > 0.05$). Causing one episode of selection to be active in these simulations can a priori be expected to cause variance in male success at that episode to be increased, because male success is linked to a trait rather than being random, thus increasing variance in reproductive success (see also [1]). However, if the two episodes are independent, whether or not selection occurs on a trait in the non-focal episode should not affect selection during the focal episode.

Table S1. Partitioning total variance in reproductive success, *T*, into copulation success, *C*, average fecundity of the female mates, *N*, and fertilization success, *F*. A subscript of *w* indicates within-pair partners and a subscript of *e* indicates extra-pair partners. All parameters were divided by $\bar{T}^2$ to enable interpretation as opportunity for selection. This simulation produced no variation in within-pair copulation success, forcing all variance and covariance terms including this parameter to equal zero (marked with an *). Following Table 1 in [6].

Variance Partition	Equation for Calculation
**Within-pair copulation success***	$\bar{N}_w^2\bar{F}_w^2Var(C_w)$
**Within-pair mate fecundity**	$\bar{C}_w^2\bar{F}_w^2Var(N_w)$
**Within-pair fertilization success**	$\bar{C}_w^2\bar{N}_w^2Var(F_w)$
**Within-pair covariance between copulation success and mate fecundity ***	$2\bar{F}_w^2\bar{C}_w\bar{N}_wCov(C_w,N_w)$
**Within-pair covariance between copulation and fertilization success***	$2\bar{N}_w^2\bar{C}_w\bar{F}_wCov(C_w,F_w)$
**Within-pair covariance between mate fecundity and fertilization success**	$2\bar{C}_w^2\bar{N}_w\bar{F}_wCov(N_w,F_w)$
**Extra-pair copulation success**	$\bar{N}_e^2\bar{F}_e^2Var(C_e)$
**Extra-pair mate fecundity**	$\bar{C}_e^2\bar{F}_e^2Var(N_e)$
**Extra-pair fertilization success**	$\bar{C}_e^2\bar{N}_e^2Var(F_e)$
**Extra-pair covariance between copulation success and mate fecundity**	$2\bar{F}_e^2\bar{C}_e\bar{N}_eCov(C_e,N_e)$
**Extra-pair covariance between copulation and fertilization success**	$2\bar{N}_e^2\bar{C}_e\bar{F}_eCov(C_e,F_e)$
**Extra-pair covariance between mate fecundity and fertilization success**	$2\bar{C}_e^2\bar{N}_e\bar{F}_eCov(N_e,F_e)$
**Covariance between within-pair and extra-pair copulation success***	$2\bar{N}_w\bar{F}_w\bar{N}_e\bar{F}_eCov(C_w,C_e)$
**Covariance between within-pair copulation success and extra-pair mate fecundity***	$2\bar{N}_w\bar{F}_w\bar{C}_e\bar{F}_eCov(C_w,N_e)$
**Covariance between within-pair copulation success and extra-pair fertilization success***	$2\bar{N}_w\bar{F}_w\bar{C}_e\bar{N}_eCov(C_w,F_e)$
**Covariance between within-pair mate fecundity and extra-pair copulation success**	$2\bar{C}_w\bar{F}_w\bar{N}_e\bar{F}_eCov(N_w,C_e)$
**Covariance between within-pair and extra-pair mate fecundity**	$2\bar{C}_w\bar{F}_w\bar{C}_e\bar{F}_eCov(N_w,N_e)$
**Covariance between within-pair mate fecundity and extra-pair fertilization success**	$2\bar{C}_w\bar{F}_w\bar{C}_e\bar{N}_eCov(N_w,F_e)$
**Covariance between within-pair fertilization success and extra-pair copulation success**	$2\bar{C}_w\bar{N}_w\bar{N}_e\bar{F}_eCov(F_w,C_e)$
**Covariance between within-pair fertilization success and extra-pair mate fecundity**	$2\bar{C}_w\bar{N}_w\bar{C}_e\bar{F}_eCov(F_w,N_e)$
**Covariance between within-pair and extra-pair fertilization success**	$2\bar{C}_w\bar{N}_w\bar{C}_e\bar{N}_eCov(F_w,F_e)$

**Error**	$Var(T) -$ sum of other components

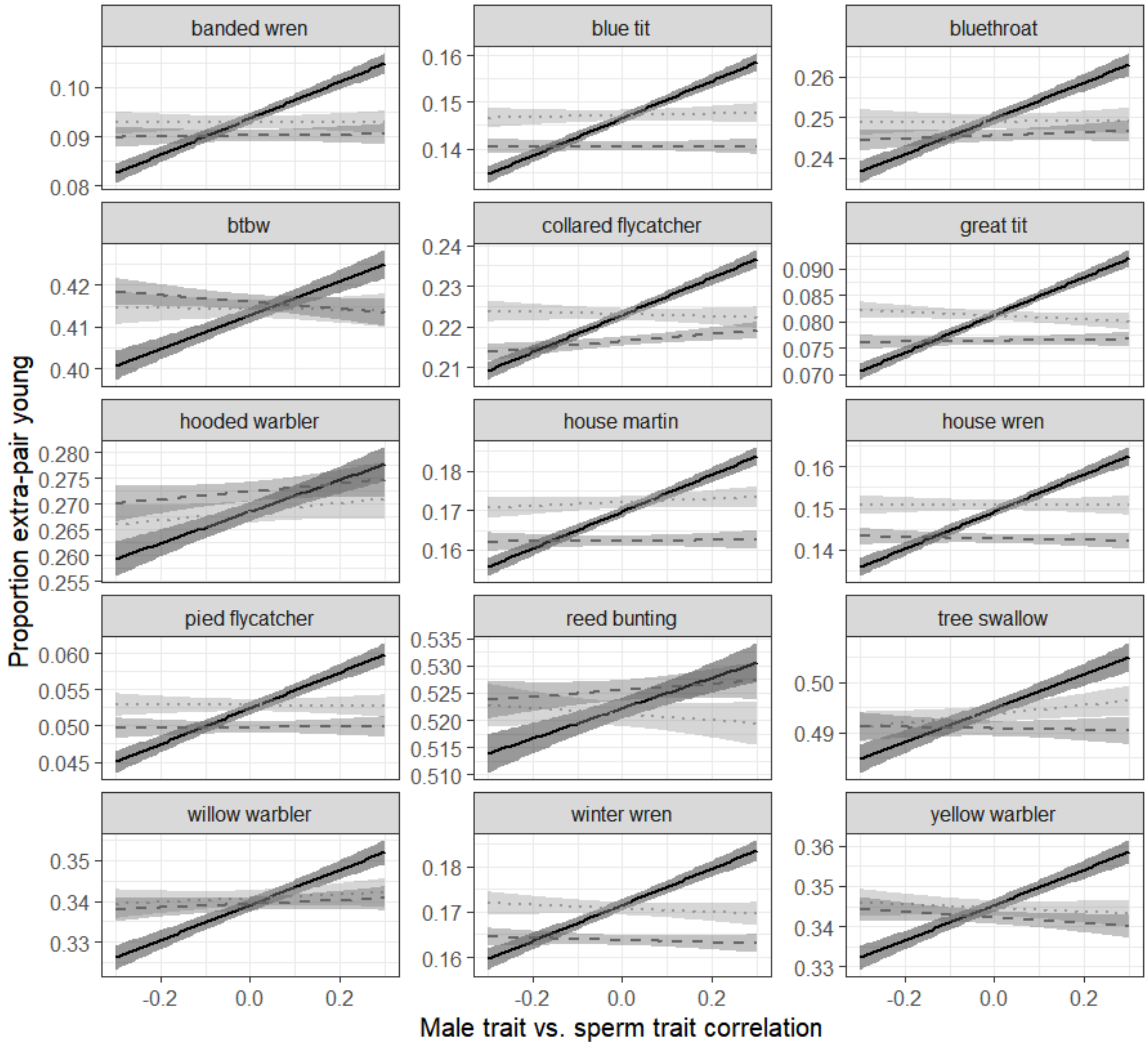

Figure S1. Proportion of extra-pair young depending on whether both traits were under selection (dark shading, solid line), only the male trait was under selection (medium shading, dashed line), or only the sperm trait was under selection (light shading, dotted line), for 15 simulated species, at various degrees of male trait vs. sperm trait correlation. Shading indicates 95% CI. Note that the y axis differs across panels, reflecting the species level variation in extra-pair paternity rates, to enable comparison with Figure 5 (see Table 1). Black-throated blue warbler is abbreviated to btbw. Minor differences in the proportion of extra-pair young across conditions appears to drive minor variation in estimated selection on the male quality trait (see Figure S2)

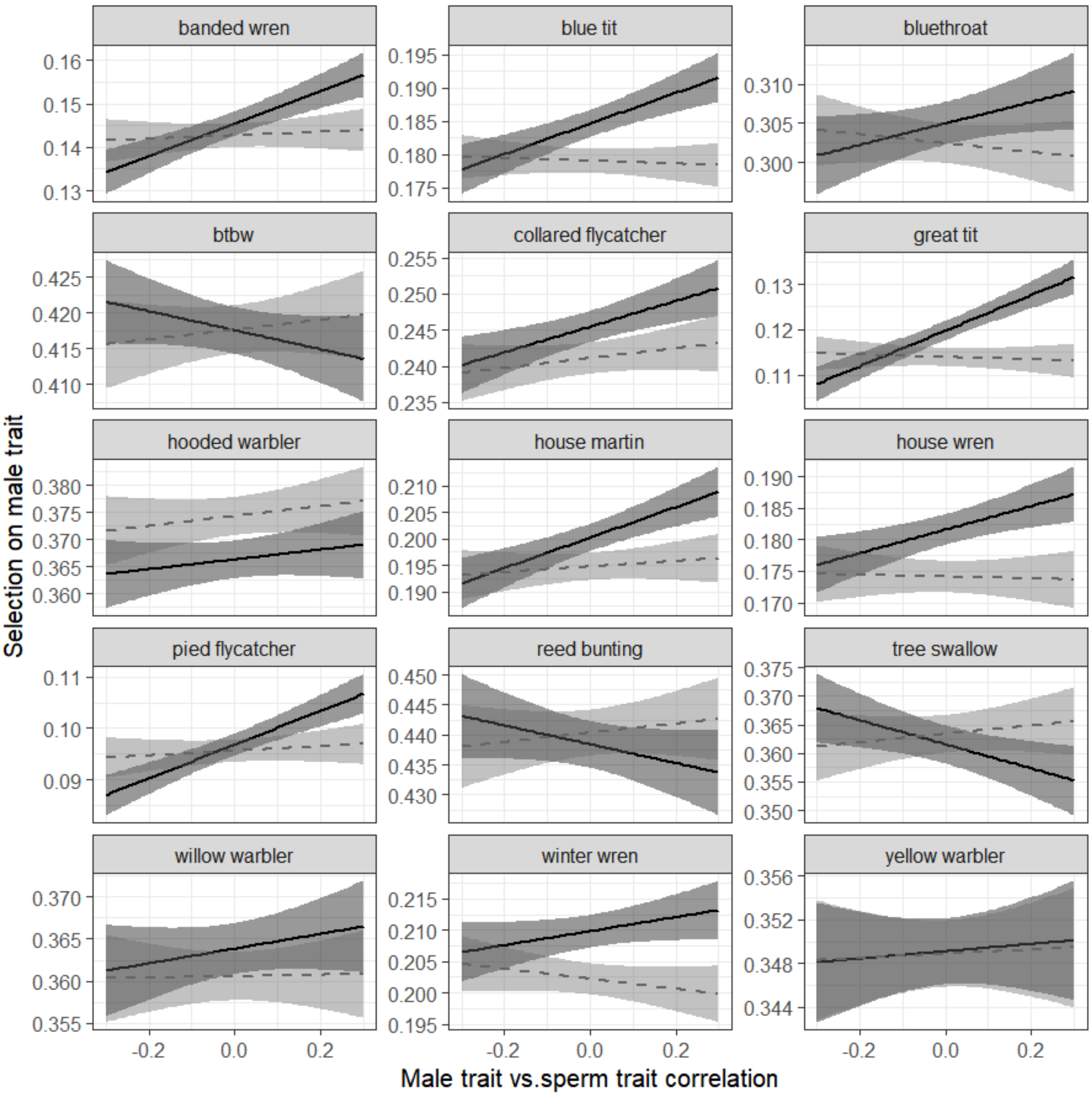

Figure S2. Strength of selection on the male trait when both episodes of selection were active (dark shading, Scheme 95. CI. Y-axis scales differ due to the differing strength of selection among species; note the relatively low absolute value of the variation within each species. The reason for the apparent decrease in selection on the male trait for the black-throated blue warbler (btbw), reed bunting, and tree swallow is unclear; within-species tests for these three species found non-significant interactions for the black-throated blue warbler and reed bunting ($F_{1,1396} < 2.7$, $p = 0.09$), although the interaction was significant for the tree swallow ($F_{1,1396} = 5.67$, $p = 0.02$).

Table S4. Estimated effect size (Cohen's *d*) for bias in parameter estimates due to correlated traits for additional simulation conditions described in the supplement. Values for the main model were re-calculated using only the correlations of -0.3 and 0.3, for direct comparability with the additional conditions (compared to values in table 2). For populations with a stronger male trait effect, only analysis results for the sperm

trait are given, and for populations with a stronger sperm trait effect, only analysis results for the male trait are given. WP = within-pair.

Analytical Approach	Focal Trait	Main Model	No Male Trait Effect WP Copulation	No Spatial Constraints	No Sperm Trait Effect on WP Fertilization	Stronger Male or Sperm Trait Effect
**Unpaired t-test: cuckolded vs. not cuckolded within-pair males**						
	Male trait	0.11	0.19	0.10	0.02	0.12
	Sperm trait	2.46	-0.03	2.47	2.60	1.38
**Unpaired t-test: males that sired vs. did not sire extra-pair offspring in other nests**						
	Male trait	0.28	0.24	0.25	0.13	0.58
	Sperm trait	0.76	0.72	0.67	0.82	2.32*
**Paired t-test: extra-pair males and the within-pair males they cuckolded**						
	Male trait	0.15	0.35	0.16	0.07	0.27
	Sperm trait	1.94	0.47	1.86	2.11	2.50*
**Univariate selection gradient: offspring produced regressed on one trait**						
	Male trait	0.26	0.43	0.27	0.05	0.37
	Sperm trait	1.33	0.37	1.29	1.55	1.70
**Multivariate selection gradient: offspring produced regressed on both traits**						
	Male trait	0.03	−0.02	0.03	−0.001	0.01
	Sperm trait	0.07	−0.02	0.04	−0.01	−0.06
**Regression of trait on detected extra-pair copulation partners**						
	Sperm trait	0.75	0.68	0.63	0.81	1.14*
**Regression of proportion of extra-pair brood sired on EP sperm trait, among broods where that male sired ≥ 1 extra-pair young**						
	Sperm trait	−0.02	−0.01	-0.01	-0.01	−0.09*
**Regression of proportion of within-pair offspring sired on WP sperm trait, among broods with ≥ 1 extra-pair young**						
	Sperm trait	0.66	−0.02	0.68	0.41	0.22

*based on 5994 populations (due to low sample size in the remaining 6)

4. Partitioning Variance in Male Reproductive Success: Results and discussion

Activating an episode of selection is expected to cause an increase in the opportunity for selection for that episode, because it increases variance in male success within that episode. This text focuses on

components involving only copulation and fertilization success; statistical results are provided for all components and for total opportunity for selection (Table S5). Figures are provided for components with reasonably high absolute values (interpreting those with low absolute values as not contributing substantially to overall opportunity for selection). Within-pair success was invariant, which is not typical in empirical studies (e.g., [8]).

When only the focal episode was active, opportunity for selection in the focal episode did not vary with the male–sperm trait correlation ($|t_{31480}| < 1.25$, $p > 0.2$, Figure S1, Table S4), except opportunity for selection on extra-pair fertilizations, which increased weakly with increasing male–sperm trait correlation ($t_{20920} = 4.48$, $p > 0.001$).

Where the male–sperm trait correlation was 0, activation of the non-focal episode did not affect opportunity for selection on within-pair fertilization success ($t = 0.70$, $p = 0.49$). In contrast, opportunity for selection on extra-pair copulation success was slightly increased, and opportunity for selection on extra-pair fertilizations was slightly decreased, by activation of the non-focal episode ($|t_{20982}| > 4.6$, $p < 0.001$, Cohen's *d* 0.02 and -0.03, respectively). Covariance between within-pair fertilization success opportunity and extra-pair copulation opportunity did not differ significantly from zero when only post-copulatory selection was active ($t_{14} = 0.14$, $p = 0.89$), but it was significantly higher (i.e., positive) when pre-copulatory selection or both episodes were active ($t_{31480} > 130$, $p < 0.001$). Covariance between within-pair and extra-pair fertilization success was positive when both episodes were active or when only post-copulatory selection was active, and was slightly higher in the latter condition ($t > 4.0$, $p < 0.01$).

When both episodes were active, opportunity for selection for all components involving only copulation and fertilization success increased with increasing male–sperm trait correlation ($t > 4.48$, $p < 0.001$; figure S3). Effect sizes were fairly low, but higher than the observed effect sizes for the number of extra-pair young (interaction term $0.06 \leq |\text{Cohen's d}| \leq 0.17$; effect sizes for number of extra-pair young, $d < 0.04$). I therefore interpret this result as partly reflecting an increase in opportunity for selection within one episode of selection, when the other episode is also active.

These results thus generally support the suggestion that opportunity for selection in one episode can strengthen or dampen opportunity for selection in the other selection episode, if success in the two episodes is positively or negatively associated, respectively [5,9]. When success in the two episodes was not correlated, differences in opportunity for selection were very slight, even if statistically significant. As the name implies, opportunity for selection gives information about the total opportunity for selection to occur, rather than the actual action of selection [10]. The relatively higher effect size for opportunity for selection, compared to realized direct selection (i.e., selection gradients from multivariate selection analysis) may illustrate that the full opportunity for selection is not reached in these simulated populations. Assessing opportunity for selection is convenient, because it is possible to do even in study systems where the traits under selection are not known [11]. However, the results of this simulation illustrate the challenge of knowing whether opportunity reflects actual selection. A novel method has recently been introduced to reduce the discrepancy between opportunity and realized selection, and should be investigated further [12].

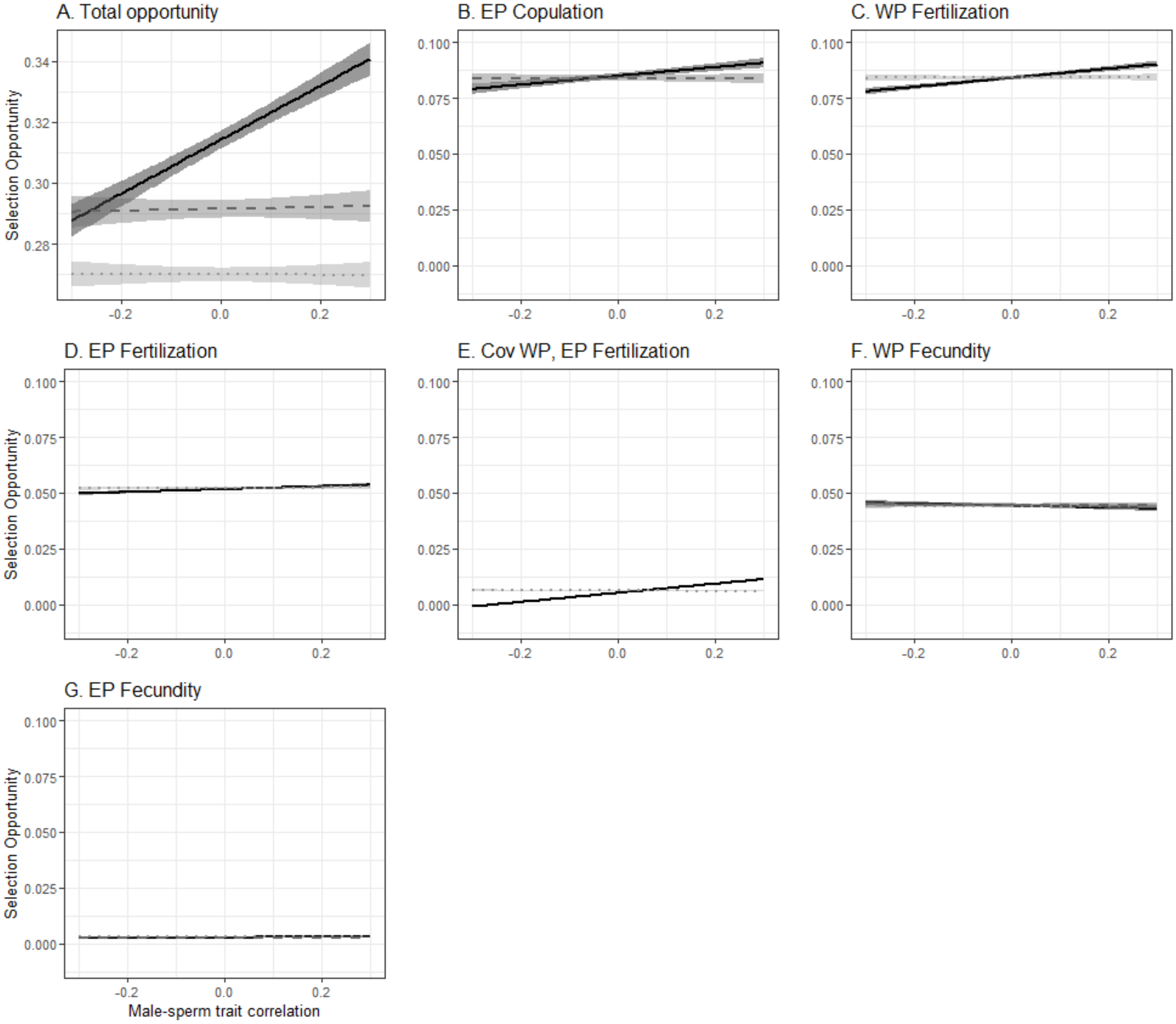

Figure S3. Opportunity for selection and its components, as a function of the male–sperm trait correlation and which episodes of selection were active. Solid, dark lines: both episodes active; medium shading, dashed line: only pre-copulatory selection active; light shading, dotted line, only post-copulatory episode active. **A**: total opportunity for selection (note different axis scale compared to other panels). **B**: Opportunity for selection on extra-pair copulations. **C**. Opportunity for selection on within-pair fertilizations. **D**. Opportunity for selection on extra-pair fertilizations. **E**. Standardized covariance between within-pair and extra-pair fertilization success. **F**. Opportunity for selection on the fecundity of the within-pair mate. **G**. Opportunity for selection on the mean fecundity of extra-pair mates.

References

1. Cramer, E.R.A.; Greig, E.I.; Kaiser, S.A. Strong sexual selection despite spatial constraints on extrapair paternity. *Behav. Ecol.* **2020**, *31*, 618–626, doi:10.1093/beheco/araa001.
2. Revell, L.J. phytools: Phylogenetic tools for comparative biology (and other things) 2016.
3. Hadfield, J.D.; Wilson, A.J.; Garant, D.; Sheldon, B.C.; Kruuk, L.E.B. The Misuse of BLUP in Ecology and Evolution. *Am. Nat.* **2010**, *175*, 116–125, doi:10.1086/648604.
4. de Villemereuil, *p.*; Nakagawa, S. General quantitative genetic methods for comparative biology. In *Modern Phylogenetic Comparative Methods and their Application in Evolutionary Biology*;

Garamszegi, L.Z., Ed.; Springer: Heidelberg, 2014; pp. 287–303.
5. Evans, J.*p*.; Garcia-Gonzalez, F. The total opportunity for sexual selection and the integration of pre- and post-mating episodes of sexual selection in a complex world. *J. Evol. Biol.* **2016**, *29*, 2338–2361, doi:10.1111/jeb.12960.
6. Webster, M.S.; Pruett-Jones, S.; Westneat, D.F.; Arnold, S.J. Measuring the effects of pairing success, extra-pair copulations and mate quality on the opportunity for sexual selection. *Evolution* **1995**, *49*, 1147–1157.
7. Arnold, S.J.; Wade, M.J. On the measurement of natural and sexual selection: applications. *Evolution* **1984**, *38*, 720–734.
8. Webster, M.S.; Tarvin, K.A.; Tuttle, E.M.; Pruett-Jones, S. Promiscuity drives sexual selection in a socially monogamous bird. *Evolution* **2007**, *61*, 2205–11, doi:10.1111/j.1558-5646.2007.00208.x.
9. Kvarnemo, C.; Simmons, L.W. Polyandry as a mediator of sexual selection before and after mating. *Philos. Trans. R. Soc. Lond. B. Biol. Sci.* **2013**, *368*, 20120042.
10. Krakauer, A.H.; Webster, M.S.; DuVal, E.H.; Jones, A.G.; Shuster, S.M. The opportunity for sexual selection: not mismeasured, just misunderstood. *J. Evol. Biol.* **2011**, *24*, 2064–71, doi:10.1111/j.1420-9101.2011.02317.x.
11. Henshaw, J.M.; Kahn, A.T.; Fritzsche, K. A rigorous comparison of sexual selection indexes via simulations of diverse mating systems. *Proc. Natl. Acad. Sci.* **2016**, *113*, E300–E308, doi:10.1073/pnas.1518067113.
12. Marie-Orleach, L.; Vellnow, N.; Schärer, L. The repeatable opportunity for selection differs between pre- and postcopulatory fitness components. *Evol. Lett.* **2021**, *5*, 101–114, doi:10.1002/evl3.210.
